# The First Vietnamese FOSD-Tacotron-2-based Text-to-Speech Model Dataset

**DOI:** 10.1016/j.dib.2020.105775

**Published:** 2020-05-27

**Authors:** Duc Chung Tran

**Affiliations:** a Computing Fundamental Department – FPT University, Hoa Lac Hi-Tech Park, Hanoi, Vietnam, 155300

**Keywords:** Text-to-speech, Natural language processing, Natural language generation, Vietnamese, Speech, Dataset, Tacotron, WaveNet

## Abstract

Recent trends in voicebot application development have enabled utilization of both speech-to-text and text-to-speech (TTS) generation techniques. In order to generate a voice response to a given speech, one needs to use a TTS engine. The recently developed TTS engines are shifting towards end-to-end approaches utilizing models such as Tacotron, Tacotron-2, WaveNet, and WaveGlow. The reason is that it enables a TTS service provider to focus on developing training and validating datasets comprising of labelled texts and recorded speeches instead of designing an entirely new model that outperforms the others which is time-consuming and costly. In this context, this work introduces the first Vietnamese FPT Open Speech Data (FOSD)-Tacotron-2-based TTS model dataset. This dataset comprises of a configuration file in *.json format; training and validating text input files (in *.csv format); a 225,000-step checkpoint of the trained model; and several sample generated audios. The published dataset is extremely worth for serving as a model for benchmarking with other newly developed TTS models / engines. In addition, it opens an entirely new TTS research optimization problem to be addressed: How to effectively generate speech from text given: a black box TTS (trained) model and its training and validation input texts.

**Specifications Table**

**Table:** 

**Subject**	Computer Science
**Specific subject area**	Artificial Intelligence; Human-Computer Interaction; Information Systems
**Type of data**	Trained model checkpoint (up to 225,000 steps) plus input training and validation datasets.
**How data were acquired**	The model was trained by utilizing Mozilla TTS repository available at [1] and the subset data (comprising of 23,000 training sentences and 1,900 validating sentences) out of over 25,000 sentences given in the FPT Open Speech Data available at [2].
**Data format**	Raw
**Parameters for data collection**	github_branch: "* master" restore_path: "" run_name: "FPTOpenSpeechData" run_description: "Tacotron2 FPTOpenSpeechData release training" audio: // Audio processing parameters num_mels: 80 num_freq: 1025 sample_rate: 22050 frame_length_ms: 50 frame_shift_ms: 12.5 preemphasis: 0.98 min_level_db: -100 ref_level_db: 20 power: 1.5 griffin_lim_iters: 60 // Normalization parameters signal_norm: true symmetric_norm: true max_norm: 4 clip_norm: true mel_fmin: 0.0 mel_fmax: 8000.0 do_trim_silence: true }, distributed: backend: "nccl", url: "tcp:\/\/localhost:54321" reinit_layers: [] model: "Tacotron2" grad_clip: 1 epochs: 1000 lr: 0.0001 lr_decay: false warmup_steps: 4000 memory_size: -1 attention_norm: "softmax" prenet_type: "original" prenet_dropout: true windowing: false use_forward_attn: false forward_attn_mask: false transition_agent: fal location_attn: true loss_masking: false enable_eos_bos_chars: falsese
	stopnet: true separate_stopnet: true tb_model_param_stats: false batch_size: 256 eval_batch_size:16 r: 7 gradual_training: [[0, 7, 32], [1, 5, 32], [50000, 3, 32], [130000, 2, 16], [290000, 1, 8]] wd: 0.000001 checkpoint: true save_step: 5000 print_step: 25 batch_group_size: 0 run_eval: true test_delay_epochs: 5 test_sentences_file: "de_sentences.txt" min_seq_len: 6->10 #default = 6; after 100000 steps: change to 10 max_seq_len: 150->100 #default = 150; after 100000 steps: change to 100 output_path: "../keep/" num_loader_workers: 4 num_val_loader_workers: 4 phoneme_cache_path: "mozilla_us_phonemes" use_phonemes: false phoneme_language: "en-us" text_cleaner: "vietnamese_cleaners" use_speaker_embedding: false style_wav_for_test: null use_gst: false datasets: name: "fptopenspeechdata" path: "/content/FPTOpenSpeechData/" meta_file_train: "metadata_train.csv" meta_file_val: "metadata_val.csv"
**Description of data collection**	This is the 1st FPT Open Speech Data (FOSD) and Tacotron-2 -based Text-to-Speech Model Dataset for Vietnamese. It comprises of: A configuration file in *.json format; Training and validation text input files (in *.csv format); A trained model (checkpoint file, after 225,000 steps); Sample generated audios. This dataset is useful for research related to TTS and its applications, text processing and especially TTS output optimization given a set of predefined input texts.
**Data source location**	Mendeley
**Data accessibility**	Tran, Duc Chung (2020), “The First FOSD-Tacotron-2-based Text-to-Speech Model Dataset for Vietnamese”, Mendeley Data, v1 [3] http://dx.doi.org/10.17632/dsmrndnmyy.1

**Value of the Data**

These data are extremely useful for benchmarking with different developed Vietnamese TTS models or engines. In addition, since input text for training and validation are provided, they open an entirely new research optimization problem to be addressed: How to effectively generate speech from text given: a black box TTS (trained) model and its training and validation input texts.
These data are useful for researches related to natural language processing, natural language generation, Vietnamese TTS applications especially for those using Artificial Intelligence and Machine Learning techniques like Tacotron, Tacotron-2, WaveGlow, WaveNet.
Those who are benefit from these data include but not limited to researchers, research scientists, students and hobbyists in the aforementioned areas, companies working in Vietnamese TTS, and automatic call centres.

## 1 Data Description

This is the 1st FPT Open Speech Data (FOSD) and Tacotron-2 -based Text-to-Speech Model Dataset for Vietnamese. It comprises of:

• A configuration file in *.json format;
• Training and validation text input files (in *.csv format);
• A trained model (checkpoint file, after 225,000 steps);
• Sample generated audios.

This dataset is useful for research related to TTS and its applications, text processing and especially TTS output optimization given a set of predefined input texts.

## 2 Experimental Design, Materials, and Methods

The following describes the experimental design, material and methods.

• Step 1: Obtain FPT Open Speech Data from [2].
• Step 2: Clean up data and transform data into format acceptable by Mozilla TTS [1]. Save cleaned data into *metadata_train.csv* (23,000 sentences) and *metadata_val.csv* (1,900 sentences).
• Step 3: Setup configuration with parameters shown in Specifications Table.
• Step 4: Train model by utilizing Mozilla TTS source code [1] and Google Colaboratory.
• Step 5: Generate audio files using the trained model with Vietnamese texts given in file *de_sentences.txt*.

## Declaration of Competing Interest

Copyright 2018 FPT Corporation Permission is hereby granted, free of charge, non-exclusive, worldwide, irrevocable, to any person obtaining a copy of this data or software and associated documentation files (the “Data or Software”), to deal in the Data or Software without restriction, including without limitation the rights to use, copy, modify, remix, transform, merge, build upon, publish, distribute and redistribute, sublicense, and/or sell copies of the Data or Software, for any purpose, even commercially, and to permit persons to whom the Data or Software is furnished to do so, subject to the following conditions: The above copyright notice, and this permission notice, and indication of any modification to the Data or Software, shall be included in all copies or substantial portions of the Data or Software. THE DATA OR SOFTWARE IS PROVIDED “AS IS”, WITHOUT WARRANTY OF ANY KIND, EXPRESS OR IMPLIED, INCLUDING BUT NOT LIMITED TO THE WARRANTIES OF MERCHANTABILITY, FITNESS FOR A PARTICULAR PURPOSE AND NONINFRINGEMENT. IN NO EVENT SHALL THE AUTHORS OR COPYRIGHT HOLDERS BE LIABLE FOR ANY CLAIM, DAMAGES OR OTHER LIABILITY, WHETHER IN AN ACTION OF CONTRACT, TORT OR OTHERWISE, ARISING FROM, OUT OF OR IN CONNECTION WITH THE DATA OR SOFTWARE OR THE USE OR OTHER DEALINGS IN THE DATA OR SOFTWARE. Patent and trademark rights are not licensed under this FPT Public License.

## Appendix Supplementary materials

Image, application 1

## References

[1] Mozilla, “Deep Learning for Text to Speech,” 2020. [Online]. Available:https://github.com/mozilla/TTS.

[2] F. T. Innovation, “30 years – FPT shares 30 hours of recorded voice data,” 2018. [Online]. Available:https://techinsight.com.vn/language/en/30-years-fpt-shares-30-hours-of-recorded-voice-data/.

[3] D. C. Tran, “The First FOSD-Tacotron-2-based Text-to-Speech Model Dataset for Vietnamese,” 2020. [Online]. Available:https://data.mendeley.com/datasets/dsmrndnmyy/1. 10.1016/j.dib.2020.105775 PMC7287261 32551347

